# Humidity-Resistive Optical NO Gas Sensor Devices Based on Cobalt Tetraphenylporphyrin Dispersed in Hydrophobic Polymer Matrix

**DOI:** 10.3390/s20051295

**Published:** 2020-02-27

**Authors:** Shunsuke Shiba, Kohei Yamada, Masanobu Matsuguchi

**Affiliations:** Department of Materials Science and Biotechnology, Graduate School of Science and Engineering, Ehime University, 3-Bunkyo-cho, Matsuyama, Ehime 790-0826, Japan; shiba.shunsuke.yu@ehime-u.ac.jp (S.S.); f844040u@mails.cc.ehime-u.ac.jp (K.Y.)

**Keywords:** metalloporphyrin, hydrophobic polymer film, optical gas sensor, nitrogen monoxide, cobalt tetraphenylporphyrin

## Abstract

We report on an optical nitrogen oxide (NO) gas sensor device using cobalt tetraphenylporphyrin (CoTPP) dispersed in three kinds of hydrophobic polymer film matrix (polystyrene (PSt), ethylcellulose (EC), and polycyclohexyl methacrylate (PCHMA)) to improve humidity resistance. Our approach is very effective because it allows us to achieve not only high humidity resistance, but also a more than sixfold increase in sensitivity compared with CoTPP film due to the high dispersion of CoTPP in the polymer film. The limit of detection was calculated as 33 ppb for the CoTPP-dispersed EC film, which is lower than that of CoTPP film (92 ppb).

## 1. Introduction

Breath analysis has recently received great attention as a noninvasive method of diagnosing several kinds of cancers [1,2,3,4], asthma [5,6], diabetes [7,8], and so on [9,10]. Conventionally, gas chromatographic separation combined with mass spectrometry (GC-MS) has been the gold standard for breath analysis because of its excellent sensitivity, limit of detection, and ability to identify molecular structures [11,12]. However, with increasing demand to improve an individual’s ability to monitor their health in daily life, such a chromatographic or mass spectrometric technique was not suitable because of its high cost and the need for both on-site analysis and high technical skills. Therefore, the development of gas sensors, which allow high-throughput on-site analysis at low cost, has grown increasingly important.

The most cumbersome obstacle in the development of gas sensors for breath analysis is the need for high selectivity. Although breath contains many kinds of gas species, including water (H_2_O), carbon monoxide (CO), carbon dioxide (CO_2_), nitrogen (N_2_), ammonia (NH_3_), nitrogen monoxide (NO), hydrogen sulfide (H_2_S), and more than 1,000 kinds of volatile organic compounds (VOC) [13], the simple molecular structures of these low molecular compounds make it difficult to achieve specific molecular recognition compared with biomarkers having complex molecular structures, such as proteins. Therefore, a recent strategy is based on chemometrics, which are comprised of pattern-recognition protocols of multiple signals obtained from sensor arrays with different selectivities [14].

To fabricate a sensor array with different selectivities, an effective strategy is to change the materials of the sensing interface. Various materials have been utilized as components of sensor arrays, including ionic liquids [15], single-stranded DNAs [16], reduced graphene oxide [17], and metalloporphyrin [14]. In particular, metalloporphyrin with different metal ligands has been preferably utilized as a sensing interface because of the ligands’ different reactivities toward gas molecules in spite of their similar structures. For example, Manno et al. reported the chemiresistive NO sensor composed of Langmuir–Blodgett films of meso, meso’-buta-1,3-diyne-bridged Cu(II) octaethylporphyrin dimer [18]. Andringa et al. reported NO gas sensor based on field-effect transistors modified with iron(III) tetraphenylporphyrin chloride [19]. Meyerhoff et al. reported optical sensors composed of indium(III) octaethylporphyrin within ultrathin polymeric films for optical sensing of amine vapors by utilizing dimer-monomer equilibrium reaction [20,21]. Tonezzer et al. reported optical sensors composed of three kinds of free, cobalt, and iron chloride 5,10,15,20 meso-tetraphenyl porphyrin, for alcoholic vapors [22]. More recently, Biring et al. took advantage of platinum(II) meso-tetrakis (pentafluorophenyl) porphyrin for oxygen sensing to develop optical dual gas sensors [23]. There are also some reports on principal component analysis (PCA). Penza et al. [24] and Liu et al. [25] reported on a metalloporphyrin-functionalized carbon nanotube-based chemiresistive gas sensor for PCA. Dunbar et al. reported on colorimetric gas sensors based on 5,10,15,20-tetrakis (3,4-bis(2-ethylhexyloxy) phenyl)-21H, 23H-porphine with six kinds of metal ligands (Mg, Sn, Zn, Au, Co, and Mn) for the PCA of volatile organic compounds (VOCs) [26]. To further increase selectivity, Chang et al. reported on a Langmuir Blodgett film consisting of a combination of supramolecular monolayers and metalloporphyrin [27]. 

5,10,15,20-Tetraphenyl-21H, 23H-porphine cobalt (II) (CoTPP) has high affinity to NO gas adsorption [28,29]. Miki et al. reported that CoTPP is a promising chemochromic material for detecting NO with excellent sensitivity and fair selectivity toward 50 ppm of CO, 5000 ppm of CO_2_, 4.8 ppm of H_2_S, and 1 ppm of NH_3_. However, the developed sensor response was significantly fluctuated by the change in humidity due to the interaction between H_2_O gas and CoTPP dispersedly adsorbed at the surface of the nonwoven porous fabrics [30]. Here, we report on optical NO gas sensor devices and propose an effective approach to increase both humidity resistance and sensitivity by combining metalloporphyrin and polymers as a matrix. The dispersion of metalloporphyrin in a polymer matrix avoids the aggregation of the porphyrin, resulting in high sensitivity derived from the increment of the adsorption sites. Although such sensitivity improvements have already been reported [30,31], it is worth noting that we could increase not only sensitivity but also humidity resistance on the basis of water solubility to the polymer matrix. For a proof-of-concept demonstration, we report here a humidity-resistive NO gas sensor by dispersing CoTPP in three kinds of hydrophobic polymers: polystyrene (PSt), ethylcellulose (EC), and polycyclohexyl methacrylate (PCHMA).

## 2. Materials and Methods

### 2.1. Materials

All chemicals were of analytical grade or better and were used as received. 5,10,15,20-Tetraphenyl-21H, 23H-porphine cobalt (II) was obtained from Sigma-Aldrich Japan. Ethyl cellulose (EC) was obtained from Kanto Chemical. PCHMA was obtained from Sigma-Aldrich Japan. Tetrahydrofuran (THF), chloroform, and polystyrene (PSt) were obtained from Fujifilm Wako Pure Chemical.

### 2.2. Fabrication of the CoTPP(-Polymer) Film

After 93.6 mg of PSt, 89.8 mg of EC, and 67.2 mg of PCHMA were each dissolved in 5 mL of tetrahydrofuran solution, 3.3 mg of CoTPP was dissolved into the prepared polymer-THF solution. The CoTPP-THF solution was also prepared in the same manner without polymer dissolution. Then, each 20 μL solution was dropped on alumina substrate and spin-coated by a spin-coater (ABLE) at 1500 rpm for 30 s in ambient air, except for the CoTPP-EC film, which was spin-coated in a N_2_ flow under a low-humidity condition (~20%RH) since its structure depends on humidity.

### 2.3. Film Characterization

Top surface structures of the fabricated films were obtained with a field emission-scanning electron microscope (FE-SEM) (S5500, Hitachi High-Technologies, Tokyo, Japan). The water droplet contact angle was measured with a contact angle meter (LSE-ME2, NiCK, Saitama, Japan). The volume of the water droplet was 0.6 μL. The optical properties were characterized by an optical system equipped with a halogen light source (MC-2563, Otsuka Electronics, Osaka, Japan) and a spectromultichannel photodetector (MSPD-7000, Otsuka Electronics, Osaka, Japan) to obtain the reflection spectrum of each film. The optical setup is detailed in Figure S1. To estimate the extent of CoTPP aggregation in the film, we obtained the reflection spectrum of each film prepared above in a N_2_ gas flow. The UV-vis transmission spectrum of 5 μM CoTPP-THF solution was also recorded by a UV-vis spectrophotometer (V-530, JASCO, Tokyo, Japan) for comparison.

### 2.4. NO Gas Sensing Properties

Optical system to apply the films to NO sensing is shown in Figure S1. The alumina substrate coated with CoTPP(-polymer) film was heated at 100 °C for the measurement of NO gas. NO gas was exposed and removed every 30 min for all the NO gas measurements. Typical responses of the CoTPP, CoTPP-EC, CoTPP-PSt, and CoTPP-PCHMA films were confirmed in flows of 10 ppm of NO gas diluted with N_2_ under low humidity (<20%RH). The humidity resistivities of the CoTPP and CoTPP-EC films were characterized by measuring 10 ppm of NO gas diluted with N_2_ under low humidity (<20%RH) twice and then the same gas composition under high humidity (85%RH) twice. Calibration curves were obtained by successive NO sensing with different concentrations; concentration was changed by controlling the flow volume ratio of NO gas and N_2_-diluted gas.

## 3. Results and Discussion

### 3.1. Structural Investigation and Hydrophobicity of the Coated Film

**Figure 1:**
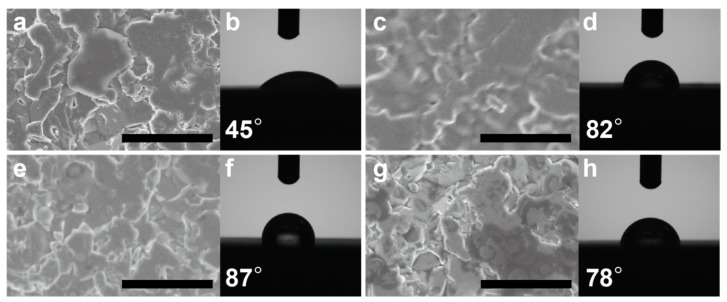
Top surface structures (**a**,**c**,**e**,**g**) and water-drop contact angle measurements (**b**,**d**,**f**,**h**) of the (**a**,**b**) cobalt tetraphenylporphyrin (CoTPP), (**c**,**d**) cobalt tetraphenylporphyrin ethylcellulose (CoTPP-EC), (**e**,**f**) cobalt tetraphenylporphyrin polystyrene (CoTPP-PSt), and (**g**,**h**) cobalt tetraphenylporphyrin polycyclohexyl methacrylate (CoTPP-PCHMA) films. Scale bar: 5 μm.

**Figure 2:**
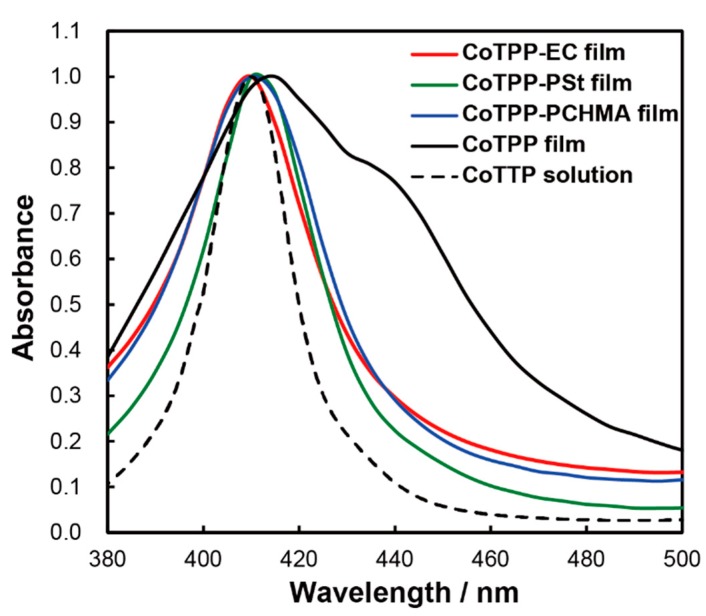
UV-vis absorbance spectra of CoTPP solution (black dotted line), CoTPP film (black solid line), CoTPP-EC film (red solid line), CoTPP-PSt film (green solid line), and CoTPP-PCHMA film (blue solid line) under nitrogen (N_2_) atmosphere.

We conducted FE-SEM observations and contact angle measurements to investigate the top surface structure and hydrophobicity of each spin-coated film. As shown in Figure 1a,c,e,g, the spin-coated films had almost the same top surface structures without remarkable pores. A micrometer-order cloud-like pattern was observed at the surface, which reflects the surface morphology of the alumina substrate below the coated films. These findings indicate that we can compare the physical and chemical properties of the film surface without considering structural differences. Figure 2b,d,f,h shows the results of the contact angle measurements of the CoTPP, CoTPP-EC, CoTPP-PSt, and CoTPP-PCHMA films. The contact angle of the CoTPP film is 45°, indicating a fairly hydrophilic surface. In contrast, the contact angles of the CoTPP-dispersed hydrophobic polymer films were greatly increased to 82°, 87°, and 78°. These results clearly suggest that the hydrophobicity of the film surfaces was successfully improved by adopting the hydrophobic polymer matrices. Note that the CoTPP molecules were evenly dispersed in each polymer matrix independent of the polymer structure, suggesting that further functionalization could additionally increase the selectivity toward other molecules.

### 3.2. Estimation of the Extent of CoTPP Aggregation

To investigate CoTPP aggregation, we obtained the UV-vis absorbance spectra of the CoTPP, CoTPP-EC, CoTPP-PSt, and CoTPP-PCHMA films as well as of the CoTPP-chloroform solution for comparison, because dissolved CoTPP molecules could be without aggregation (the spectra with more wide wavelength window including Q-band are shown in Figure S2). We normalized the absorbance by the peak intensity at around 410 nm. The CoTPP solution showed a sharp peak with a full width at half maximum (FWHM) of 21 nm (Figure 2, black dotted line). Compared with the CoTPP solution, the CoTPP film showed two peaks, one at 410 nm and another around 440 nm, and the resultant FWHM was much wider (70 nm). These results indicate that the CoTPP molecules were greatly aggregated with each other. In contrast, the FWHMs obtained from the CoTPP-EC (35 nm), CoTPP-PSt (30 nm), and CoTPP-PCHMA films (40 nm) became much narrower than that of the CoTPP film, and each polymer film without CoTPP exhibited no noticeable peaks as shown in Figure S1. These results indicate that the existence of the polymer matrix successfully prevented the CoTPP from aggregating.

### 3.3. Sensor Responses to NO Gas

**Figure 3:**
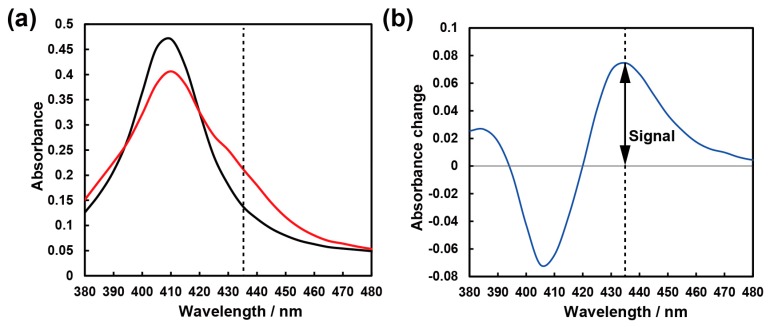
(**a**) Absorption spectra of the CoTPP-EC film before and after exposure to 10 ppm nitrogen oxide (NO)/N_2_ gas in low humidity (~0%RH). (**b**) Subtraction of the absorption spectra before and after exposure to 10 ppm NO/N_2_ gas.

Figure 3a shows the typical absorption spectral change before and after NO gas exposure. When NO molecules coordinate to the cobalt ligand in a porphyrin ring, the oxidation states are transferred from Co(II) to Co(III), bringing about a change in the adsorption spectra around the Soret band (380–480 nm). As shown in Figure 3b, the subtraction of the absorption spectra shows a maximum value at the wavelength of 435 nm. Therefore, we defined the signal as the absorbance change at 435 nm because the variation of absorbance is largest at this wavelength.

**Figure 4:**
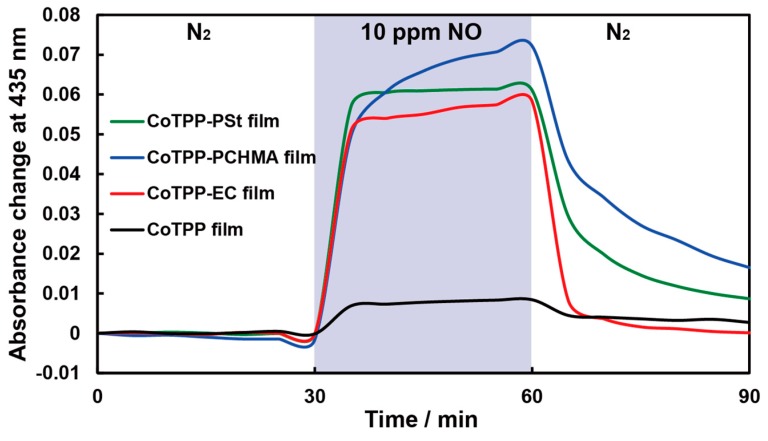
Typical responses of CoTPP film (black), CoTPP-EC film (red), CoTPP-PCHMA film (green), and CoTPP-PSt film to 10 ppm of NO/N_2_ at 100 °C. The blue zone indicates the period of NO gas introduction.

Next, we investigated the effect of suppressing CoTPP aggregation on the sensitivity of optical NO sensing. As shown in Figure 4, the CoTPP-polymer films exhibit 7–8 times larger signals for 10 ppm NO gas than the CoTPP film, indicating that NO gas could enter the polymer and change the spectra of the CoTPP molecules. In other words, the insertion of NO molecules into the CoTPP films was difficult because of the strong stacking of CoTPP molecules. Interestingly, the recovery of the CoTPP-EC film was faster than those of the CoTPP-PSt and CoTPP-PCHMA films even though they had almost the same sensitivity. This is probably due to the difference in film thicknesses. Therefore, we focused on the CoTPP-EC film and the CoTPP film to further investigate the gas sensing properties.

However, about 5 min was required to reach equilibrium state after introducing NO gas into the gas chamber, which is a significantly longer time than that previously reported, where the CoTPP molecules adsorb at the surface of nonwoven fabrics, resulting in fast response and low water resistivity [30]. These slow responses and recoveries may be derived from the slow diffusion process of NO gas into each polymer matrix. Therefore, optimization of the film thickness and porosity may be very important for further improving the performance of the polymer-CoTPP film.

### 3.4. Humidity Dependence of NO Sensitivity

**Figure 5:**
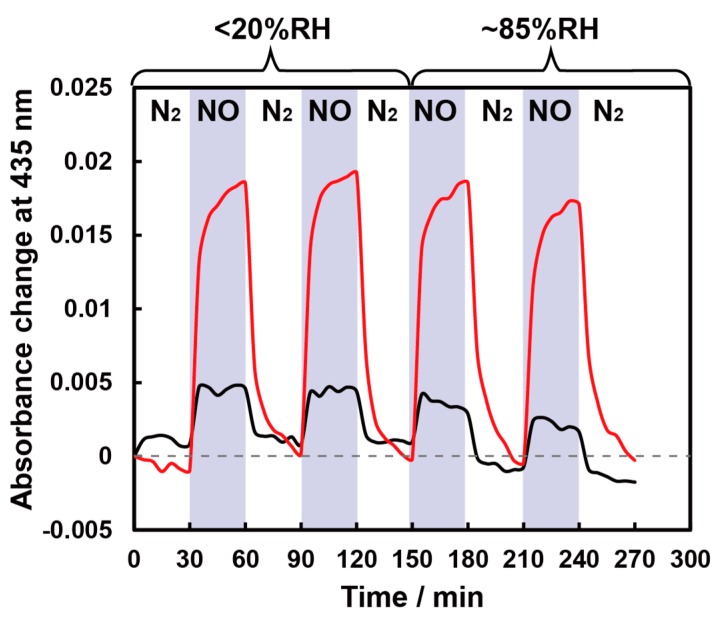
One ppm NO sensing at 100 °C under low humidity (<20 RH%) and high humidity (85 RH%) conditions for CoTPP-EC film (red solid line) and CoTPP film (black solid line). The blue zone indicates the period of NO gas introduction.

We then confirmed the dependence of the NO sensitivity on humidity. Since the relative humidity (RH) of the expiration is around 90%RH, we adopted a relative humidity of 85 RH%. As shown in Figure 5, the signal of the CoTPP film drifted under high humidity. This tendency is expected to be more significant when the NO concentration is at the ppb-level, which has to be detected for applications such as diagnosing asthma. In contrast, no such drift was observed with the hydrophobic CoTPP-EC film, the sensitivity of which was very high. This clearly indicates that we successfully increased the resistivity toward H_2_O gas. It is worth repeating that our approach, based on the simple drop-casting technique, enables us to improve both sensitivity and selectivity effectively.

### 3.5. Calibration Curves

**Figure 6:**
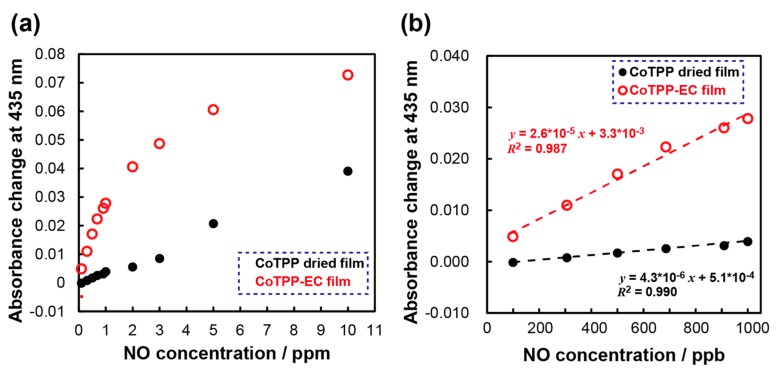
(**a)** Calibration curves of NO gas obtained with CoTPP film (black) and CoTPP-EC film. (**b**) Magnified curves in the concentration range of 50 to 1000 ppb.

Figure 6a shows the calibration curves obtained for the CoTPP and CoTPP-EC films. For the CoTPP film, the signal increased linearly with the NO concentration, but the sensitivity decreased (4.3 × 10^−6^ ppb^−1^). On the other hand, the CoTPP-EC film showed high sensitivity in the low-concentration region but exhibited a nonlinear relationship especially in the high-concentration region. This nonlinear relationship is reasonable, not only because of the NO adsorption to the CoTPP but also because of the absorption into the polymer involved in the total response. This was not the case with the CoTPP film, because CoTPP stacking may have blocked the absorption into CoTPP film, as suggested by the resultant low sensitivity. Figure 6b shows magnified calibration curves in the region of 0 to 1 ppm in Figure 6a. If the curve at low concentration is regarded as linear, the sensitivity estimated from the slope of CoTPP-EC film was 2.6 × 10^−5^ ppb^−1^, which is sixfold that of the CoTPP film. This sensitivity enhancement is in agreement with the results of Figure 4. The limit of detection (LOD) for the CoTPP-EC film was calculated as 33 ppb (S/N = 3), which is lower than that of CoTPP (92 ppb). Since repeated measurements demonstrated that the CoTPP-EC film exhibited higher stability for NO detection (RSD: 3.1%, n = 10), as shown in Figure S3, the developed CoTPP-EC film can be utilized as the sensing interface for both disposable and nondisposable gas sensor devices.

## 4. Conclusions

Optical NO sensor devices composed of CoTPP dispersed in three kinds of hydrophobic polymer film were developed. In addition to the intrinsic sensitivity and selectivity of CoTPP toward NO gas, the existence of a hydrophobic polymer matrix resulted not only in greater resistance to humidity resistivity but also in a more than six times increase in sensitivity thanks to the suppression CoTPP aggregation. The limit of detection of NO gas obtained by the CoTPP-EC film was 33 ppb, which is lower than that obtained by the CoTPP film (92 ppb). Our proposed approach might be very versatile and applicable not only to humidity resistance but also to that of any other molecule by designing a polymer structure. Since these films were fabricated by a simple drop-casting technique, we believe that the printing technique is applicable to scalable fabrication for sensor arrays and could expand to PCA for practical applications.

## Supplementary Materials

The following are available online at https://www.mdpi.com/1424-8220/20/5/1295/s1, Figure S1: Block diagram of the optical system. Figure S2: UV-vis absorbance spectra of (a) EC film, (b) PSt film, and (c) PCHMA film with or without CoTPP. Figure S3: Stability test of CoTPP-EC film.

## References

[1] Amor R.E., Nakhleh M.K., Barash O., Haick H. (2019). Breath analysis of cancer in the present and the future. Eur. Respir. Rev..

[2] Markar S.R., Wiggins T., Antonowicz S., Chin S.T., Romano A., Nikolic K., Evans B., Cunningham D., Mughal M., Lagergren J. (2018). Assessment of a noninvasive exhaled breath test for the diagnosis of oesophagogastric cancer. JAMA Oncol..

[3] Durán-Acevedo C.M., Jaimes-Mogollón A.L., Gualdrón-Guerrero O.E., Welearegay T.G., Martinez-Marín J.D., Caceres-Tarazona J.M., Sánchez-Acevedo Z.C., Beleño-Saenz K.J., Cindemir U., österlund L. (2018). Exhaled breath analysis for gastric cancer diagnosis in Colombian patients. Oncotarget.

[4] Chang J.E., Lee D.S., Ban S.W., Oh J., Jung M.Y., Kim S.H., Park S., Persaud K., Jheon S. (2018). Analysis of volatile organic compounds in exhaled breath for lung cancer diagnosis using a sensor system. Sens. Actuators B Chem..

[5] Ibrahim B., Basanta M., Cadden P., Singh D., Douce D., Woodcock A., Fowler S.J. (2011). Non-invasive phenotyping using exhaled volatile organic compounds in asthma. Thorax.

[6] Schneider A., Tilemann L., Schermer T., Gindner L., Laux G., Szecsenyi J., Meyer F.J. (2009). Diagnosing asthma in general practice with portable exhaled nitric oxide measurement-results of a prospective diagnostic study. Respir. Res..

[7] Rydosz A. (2018). Sensors for enhanced detection of acetone as a potential tool for noninvasive diabetes monitoring. Sensors.

[8] Sachdeva S., Agarwal R., Agarwal A. (2019). MEMS based tin oxide thin film gas sensor for diabetes mellitus applications. Microsyst. Technol..

[9] Kabir E., Raza N., Kumar V., Singh J., Tsang Y.F., Lim D.K., Szulejko J.E., Kim K.H. (2019). Recent Advances in Nanomaterial-Based Human Breath Analytical Technology for Clinical Diagnosis and the Way Forward. Chem..

[10] Zajda J., Schmidt N.J., Zheng Z., Wang X., Meyerhoff M.E. (2018). Performance of Amperometric Platinized—Nafion Based Gas Phase Sensor for Determining Nitric Oxide (NO) Levels in Exhaled Human Nasal Breath. Electroanalysis.

[11] Mleth M., Schubert J.K., Gröger T., Sabei B., Kischkel S., Fuchs P., Hein D., Zimmermann R., Miekisch W. (2010). Automated needle trap heart-cut GC/MS and needle trap comprehensive two-dimensional GC/TOF-MS for breath gas analysis in the clinical environment. Anal. Chem..

[12] Wang X.R., Cassells J., Berna A.Z. (2018). Stability control for breath analysis using GC-MS. J. Chromatogr. B.

[13] Kim K.H., Jahan S.A., Kabir E. (2012). A review of breath analysis for diagnosis of human health. TrAC Trends Anal. Chem..

[14] Feng S., Farha F., Li Q., Wan Y., Xu Y., Zhang T., Ning H. (2019). Review on smart gas sensing technology. Sensors.

[15] Park C.H., Schroeder V., Kim B.J., Swager T.M. (2018). Ionic Liquid-Carbon Nanotube Sensor Arrays for Human Breath Related Volatile Organic Compounds. ACS Sens..

[16] Jung Y., Moon H.G., Lim C., Choi K., Song H.S., Bae S., Kim S.M., Seo M., Lee T., Lee S. (2017). Humidity-Tolerant Single-Stranded DNA-Functionalized Graphene Probe for Medical Applications of Exhaled Breath Analysis. Adv. Funct. Mater..

[17] Alizadeh T., Soltani L.H. (2016). Reduced graphene oxide-based gas sensor array for pattern recognition of DMMP vapor. Sens. Actuators B Chem..

[18] Manno D., Micocci G., Serra A., Tepore A., Valli L., Arnold D.P. (1999). Gas sensing properties of meso, meso′-buta-1,3-diyne-bridged Cu(II) octaethylporphyrin dimer Langmuir-Blodgett films. Sens. Actuators B Chem..

[19] Andringa A.M., Spijkman M.J., Smits E.C.P., Mathijssen S.G.J., Hal P.A.v., Setayesh S., Willard N.P., Borshchev O.V., Ponomarenko S.A., Blom P.W.M. (2010). Gas sensing with self-assembled monolayer field-effect transistors. Org. Electron..

[20] Qin W., Parzuchowski P., Zhang W., Meyerhoff M.E. (2003). Optical sensor for amine vapors based on dimer-monomer equilibrium of indium(III) octaethylporphyrin in a polymeric film. Anal. Chem..

[21] Kang Y., Meyerhoff M.E. (2006). Rapid response optical ion/gas sensors using dimer-monomer metalloporphyrin equilibrium in ultrathin polymeric films coated on waveguides. Anal. Chim. Acta.

[22] Tonezzer M., Quaranta A., Maggioni G., Carturan S., Mea G.D. (2007). Optical sensing responses of tetraphenyl porphyrins toward alcohol vapours: A comparison between vacuum evaporated and spin-coated thin films. Sens. Actuators B Chem..

[23] Biring S., Sadhu A.S., Deb M. (2019). An effective optical dual gas sensor for simultaneous detection of oxygen and ammonia. Sensors.

[24] Penza M., Rossi R., Alvisi M., Signore M.A., Serra E., Paolesse R., D’Amico A., Di Natale C. (2010). Metalloporphyrins-modified carbon nanotubes networked films-based chemical sensors for enhanced gas sensitivity. Sens. Actuators B Chem..

[25] Liu S.F., Moh L.C.H., Swager T.M. (2015). Single-walled carbon nanotube-metalloporphyrin chemiresistive gas sensor arrays for volatile organic compounds. Chem. Mater..

[26] Dunbar A.D.F., Brittle S., Richardson T.H., Hutchinson J., Hunter C.A. (2010). Detection of volatile organic compounds using porphyrin derivatives. J. Phys. Chem. B.

[27] Lu Y., Chang Y., Tang N., Qu H., Liu J., Pang W., Zhang H., Zhang D., Duan X. (2015). Detection of Volatile Organic Compounds Using Microfabricated Resonator Array Functionalized with Supramolecular Monolayers. ACS Appl. Mater. Interfaces.

[28] Okajima T., Yamamoto Y., Ouchi Y., Seki K. (2001). NEXAFS spectra of metallotetraphenylporphyrins with adsorbed nitrogen monoxide. J. Electron. Spectrosc. Relat. Phenom..

[29] Miyamoto M., Hanazato Y. (1998). Nitrogen Monoxide Adsorption and Contact Decomposition Properties of Co(II) Complexes. Nippon Kagaku Kaishi.

[30] Miki H., Matsubara F., Nakashima S., Ochi S., Nakagawa K., Matsuguchi M., Sadaoka Y. (2016). A fractional exhaled nitric oxide sensor based on optical absorption of cobalt tetraphenylporphyrin derivatives. Sens. Actuators B Chem..

[31] Itagaki Y., Yamanaka S., Sadaoka Y. (2011). HCl detection using polymer-porphyrin composite coated optical fiber sensor. Sens. Lett..

