# Supplementary material for: Humidity-Resistive Optical NO Gas Sensor Devices Based on Cobalt Tetraphenylporphyrin Dispersed in Hydrophobic Polymer Matrix
Source: Sensors (Basel). 2020 Feb 27;20(5):1295. doi: 10.3390/s20051295 (PMC7085509; doi:10.3390/s20051295)
Supplement: Supplementary file 1 [file sensors-20-01295-s001.pdf]

Supporting Information

Humidity-Resistive Optical NO Gas Sensor Devices Based on the Cobalt Tetraphenylporphyrin Dispersed in Hydrophobic Polymer Matrix

Shunsuke Shiba, Kohei Yamada and Masanobu Matsuguchi *

Department of Materials Science and Biotechnology, Graduate School of Science and Engineering, Ehime University, 3-Bunkyo-cho, Matsuyama, Ehime 790-0826, Japan; shiba.shunsuke.yu@ehime-u.ac.jp
* Correspondence: matsuguchi@ehime-u.ac.jp; Tel.: +81-89-927-9933

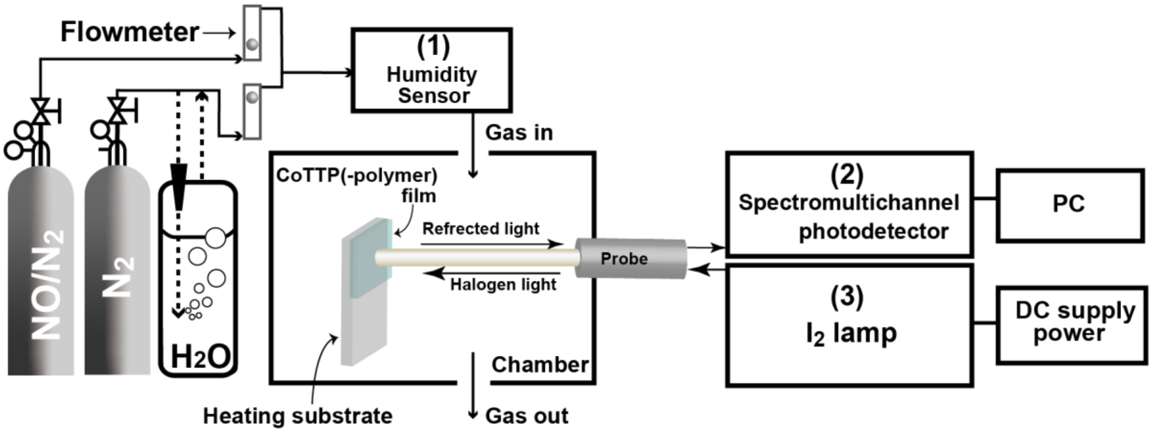

Figure S1. Block diagram of the optical system equipped with (1) separate type humidity sensor (HTY7843, Yamatake), (2) Spectromultichannel photodetector MSPD-7000; Otsuka Electronics), (3) I_2 lamp (MC-2563; Otsuka Electronics), and so on. Dotted arrows indicate the gas flow root in case of requiring high humidity.

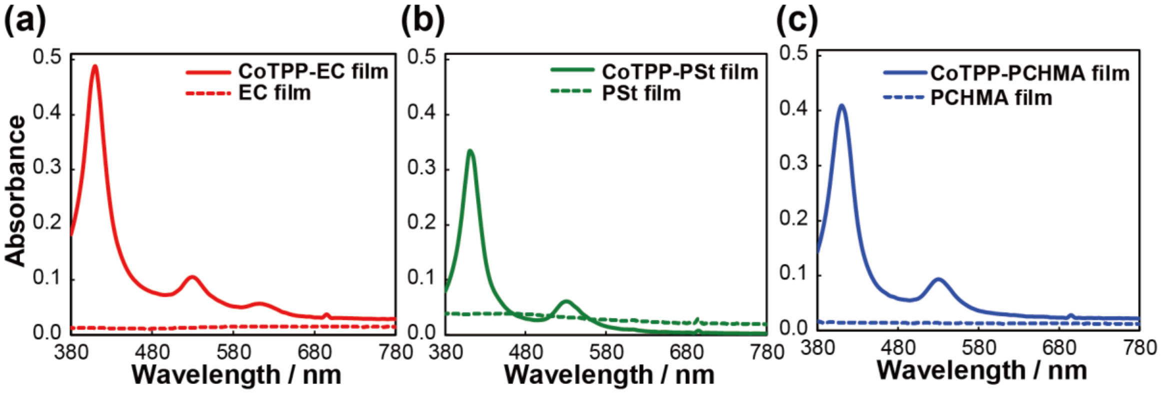

Figure S2. UV-vis absorbance spectra of (a) EC film (red), (b) PSt film (green), and (c) PCHMA film (blue) with (solid line) or without (dotted line) CoTPP.

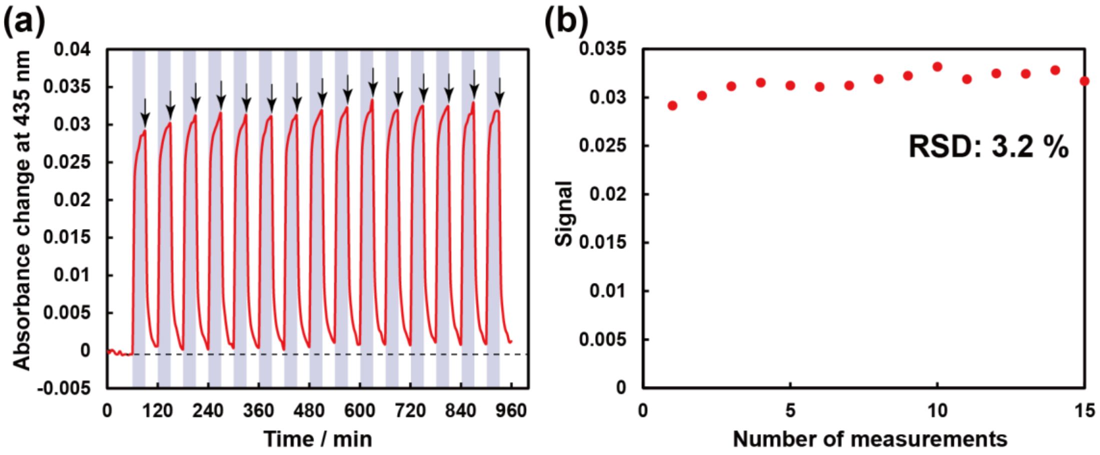

Figure S3. Stability test of CoTPP-EC film. **(a)** Repeated measurements of 1 ppm NO/N_2. The blue zone indicates the periods of NO gas introduction. Dotted line and arrows indicate the baseline and the point 30 min after introducing NO gas. **(b)** Data plots of the signals calculated from (a) on the number of measurements. Signal was calculated by subtracting baseline from the values of absorbance change indicated by arrows in (a).
